# Incretin-Based Therapies and Post–Bariatric Surgery Alcohol Use Disorder

**DOI:** 10.1001/jamanetworkopen.2025.49086

**Published:** 2025-12-22

**Authors:** Butros Fakhoury, Leandro Sierra, Kaanthi Rama, Vinay Jahagirdar, Luis Antonio Díaz, Juan Pablo Arab

**Affiliations:** 1 Department of Medicine, Virginia Commonwealth University School of Medicine, Richmond; 2 Department of Medicine, Cleveland Clinic Foundation, Cleveland, Ohio; 3 Division of Gastroenterology, Hepatology, and Nutrition, Department of Internal Medicine, Virginia Commonwealth University School of Medicine, Richmond; 4 Departamento de Gastroenterología, Escuela de Medicina, Pontificia Universidad Católica de Chile, Santiago, Chile; 5 MASLD Research Center, Division of Gastroenterology and Hepatology, University of California San Diego, La Jolla

## Abstract

**Importance:**

Patients who have undergone bariatric surgery have an elevated risk for alcohol use disorder (AUD). Incretin-based therapies (IBTs) may be associated with reward pathways in addition to weight loss.

**Objective:**

To evaluate whether IBT after bariatric surgery is associated with a lower risk of new-onset AUD and initiation of medications for AUD (MAUDs) compared with non-IBT antiobesity medications (AOMs).

**Design, Setting, and Participants:**

This retrospective cohort study included 15 382 adults who underwent bariatric surgery and subsequently received an AOM between January 1, 2020, and January 1, 2024, with outcomes assessed up to 2 years after AOM initiation. Data were derived from a multi-institutional US electronic health record network. Propensity score matching (1:1) balanced baseline covariates. Data were analyzed September 14, 2025.

**Exposures:**

Post–bariatric surgery treatment with an IBT (semaglutide, liraglutide, or tirzepatide) vs non-IBT AOMs (orlistat, phentermine, low-dose naltrexone, benzphetamine, phendimetrazine, or diethylpropion).

**Main Outcomes and Measures:**

Outcomes of interest were incidence rates (per 1000 person-years) and hazard ratios (HRs) of new-onset AUD and initiation of MAUDs, estimated using Kaplan-Meier and Cox proportional hazards regression models.

**Results:**

The study included 15 382 patients who underwent bariatric surgery and subsequently received AOMs (11 194 IBT [mean (SD) age, 51.4 (11.6) years; 8855 women (79.1%)]; and 4188 non-IBT [mean (SD) age, 45.1 (11.0) years; 3587 women (86.6%)]). After propensity score matching, 3990 patients were included in each group. Use of IBT was associated with a lower incidence of AUD (2.4 vs 5.2 per 1000 person-years) and a lower hazard of developing AUD (HR, 0.45; 95% CI, 0.25-0.81; *P* = .006) vs non-IBT use. Use of IBT was also associated with a lower incidence of initiating MAUDs (15.2 vs 25.6 per 1000 person-years) and a lower hazard of MAUD initiation (HR, 0.59; 95% CI, 0.46-0.75; *P* < .001). Results were consistent across sensitivity analyses, including restriction to AOM initiation within 5 years of bariatric surgery and requiring 3 or more AOM prescriptions.

**Conclusions and Relevance:**

In this cohort study of patients who underwent bariatric surgery, IBT was associated with a 55% lower risk of new-onset AUD and a 41% lower risk of initiation of MAUDs compared with non-IBT AOMs. These findings suggest potential neurobehavioral benefits associated with IBTs that may inform AOM selection in this high-risk population. Prospective studies are warranted to confirm these associations and examine long-term liver-related outcomes.

## Key Points

**Question:**

Is the use of incretin-based therapies (IBTs) after bariatric surgery associated with a reduced risk of alcohol use disorder (AUD) compared with non-IBT antiobesity medications?

**Findings:**

In this multi-institutional propensity-matched cohort study of nearly 8000 patients who had undergone bariatric surgery, IBT was associated with a 55% lower risk of new-onset AUD and a 41% lower risk of initiating medications for AUD, with consistent results across multiple sensitivity analyses.

**Meaning:**

This study found that IBTs may be associated with neurobehavioral benefits in addition to weight loss, suggesting that they should be prioritized for patients who had undergone bariatric surgery and are at risk for AUD.

## Teaser

This cohort study evaluates whether incretin-based therapy after bariatric surgery is associated with a lower risk of new-onset alcohol use disorder and initiation of medications for alcohol use disorder compared with non–incretin-based therapy antiobesity medications.

## Introduction

Obesity has become a global epidemic and is a major risk factor for cardiovascular disease, metabolic dysfunction–associated steatotic liver disease, chronic kidney disease, and all-cause mortality.^1^ Over 2 billion adults worldwide are overweight or obese, and the global prevalence continues to increase substantially.^2^ Bariatric surgery remains one of the most effective interventions for sustained weight loss.^3^ However, emerging data suggest it may paradoxically increase the risk of developing addictive behaviors, including alcohol use disorder (AUD).^4,5,6,7,8^ This phenomenon, often described as “addiction transfer,” is thought to result from shared psychological and neurobiological pathways that regulate hedonic eating and substance use, potentially amplified by altered gut hormone signaling and accelerated alcohol absorption.^8^ According to a position statement published by the American Society for Metabolic and Bariatric Surgery, AUD is considered a relative contraindication to bariatric surgery, and patients undergoing bariatric surgery should be screened and counseled regarding alcohol use both before and after the procedure.^9^

Incretin-based therapies (IBTs), including glucagon-like peptide-1 receptor agonists (GLP-1RAs) and their combination with glucose-dependent insulinotropic polypeptide (GIP) and/or glucagon receptor agonists, exert metabolic effects through both peripheral and central mechanisms, enhancing glycemic control and promoting weight loss.^10^ Several agents, such as semaglutide (GLP-1RA) and tirzepatide (dual GLP-1 and GIP receptor agonists), have emerged as effective antiobesity medications (AOMs) and are frequently used as adjunctive therapy for patients who underwent bariatric surgery and experienced weight regain or inadequate weight loss.^11,12^ Preclinical studies suggest that IBTs attenuate the ability of alcohol to activate the mesolimbic dopamine system and reduce alcohol-seeking behaviors.^13,14^ Clinical data also support a potential role for IBTs in reducing alcohol consumption.^15,16,17,18^ Although prior studies have examined the association of bariatric surgery and IBTs with AUD independently, to our knowledge, no study has directly evaluated the association of IBT with AUD risk in patients at high risk who have undergone bariatric surgery. Thus, we conducted a large, multi-institutional cohort study to evaluate whether treatment with IBTs is associated with a reduced risk of AUD and initiation of medications for AUD (MAUDs) after bariatric surgery.

## Methods

### Study Design and Data Source

This retrospective cohort study used data from TriNetX (Cambridge, Massachusetts), a federated health research platform that provides real-time access to deidentified electronic health records from included health care organizations. The TriNetX US Collaborative Network, which aggregates patient-level data across more than 70 US health care organizations, was used for this analysis. The dataset includes patient demographic characteristics; diagnoses (*International Statistical Classification of Diseases and Related Health Problems, Tenth Revision* [*ICD-10*] and *International Statistical Classification of Diseases, Tenth Revision, Clinical Modification* [*ICD-10-CM*]); procedures (*ICD-10* Procedure Coding System and *Current Procedural Terminology* codes); medications (Veterans Affairs Drug Classification System and RxNorm); and laboratory test values (Logical Observation Identifiers Names and Codes). Data are verified and quality checked prior to extraction from source electronic health records and integration into the TriNetX platform. Complete data from all participating sites in the US Collaborative Network were extracted on September 14, 2025. The study was deemed exempt from review by the Virginia Commonwealth University institutional review board, as it used only data that were deidentified. Patient informed consent was not required for this study as it used only deidentified, aggregate-level data. This study was conducted per Good Clinical Practice guidelines, the Declaration of Helsinki,^19^ and applicable local laws. The study also followed the Strengthening the Reporting of Observational Studies in Epidemiology (STROBE) reporting guidelines.

### Cohort Selection

Cohort selection and inclusion and exclusion criteria are shown in eFigure 1 in Supplement 1. The cohort included adult patients (aged ≥18 years) undergoing bariatric surgery who received AOMs between January 1, 2020, and January 1, 2024. Bariatric surgery included Roux-en-Y gastric bypass and sleeve gastrectomy, identified using a validated coding algorithm supplemented by *ICD-10-CM* code Z98.84 (bariatric surgery status) and a body mass index (BMI; calculated as weight in kilograms divided by height in meters squared) of 35 or more to improve specificity.^20^ Antiobesity medications included semaglutide, liraglutide, tirzepatide, orlistat, phentermine, naltrexone (<8 mg), benzphetamine, phendimetrazine, and diethylpropion.

Patients were excluded if they had a preexisting diagnosis of AUD prior to AOM initiation or if they received both IBT and non-IBT medications during follow-up. Combination medications such as phentermine-topiramate and naltrexone-bupropion are coded as separate components in the dataset (eg, phentermine and topiramate individually), rather than as fixed-dose combinations. To minimize misclassification bias, we excluded topiramate and bupropion from the analysis. Patients who received only low-dose naltrexone (<8 mg) prior to the index date without any prescription of higher dosages were included in the non-IBT group to minimize misclassification bias, given naltrexone’s dual indication as both an AOM and a MAUD.^21^ Patients were stratified into IBT (semaglutide, liraglutide, or tirzepatide) and non-IBT (orlistat, phentermine, naltrexone [<8 mg], benzphetamine, phendimetrazine, and diethylpropion) groups (eTable 1 in Supplement 1).

### Covariates and Definitions

Demographic characteristics (age, sex, and race and ethnicity [Asian, Black or African American, Hispanic or Latino, White, and other [defined internally by TriNetX and includes American Indian or Alaska Native, Native Hawaiian or Other Pacific Islander, and all other nonspecified race or ethnic group categories]) and socioeconomic and psychosocial factors were obtained from the electronic health record as documented at the time of the clinical encounter. Race and ethnicity were collected to describe the cohort and assess demographic differences in alcohol-related outcomes after bariatric surgery. Cardiometabolic comorbidities (type 2 diabetes, hypertension, and obesity) were incorporated as proxies for medical comorbidities, liver disease comorbidities were incorporated as proxies for alcohol exposure, and baseline liver enzyme levels were incorporated as proxies for liver-related harm. Psychiatric and substance use disorders (depression, anxiety, and cannabis-, opioid-, stimulant-, and sedative-related disorders) were included for their relevance to AUD management and prognosis. Medications with recognized off-label use in the management of AUD (baclofen, topiramate, and gabapentin) were included to account for potential pharmacologic confounding (eTable 2 in Supplement 1).

### Outcomes and Definitions

The primary outcome was a new diagnosis of AUD, defined by *ICD-10-CM* codes for alcohol abuse or dependence as previously described in the literature (eTable 1 in Supplement 1).^22^ Nonspecific *ICD-10-CM* codes (eg, F10.9) were excluded to reduce misclassification. The secondary outcome was the prescription of MAUDs (naltrexone [≥50 mg], acamprosate, or disulfiram). The index date was defined as the date of first AOM prescription after bariatric surgery. To allow sufficient exposure, a 1-month lag period after the index AOM prescription was applied before outcome assessment. The follow-up period was limited to 2 years to ensure a comparable follow-up period across groups, given the more recent approval of IBTs.

### Statistical Analysis

Statistical analysis was conducted September 14, 2025. Categorical variables were summarized as frequencies and percentages and compared using Pearson χ^2^ tests, while continuous variables were presented as mean (SD) values and compared using independent-sample *t* tests (eTable 3 in Supplement 1). Propensity score matching (PSM) was performed using logistic regression based on the previously mentioned covariates. One-to-one greedy nearest-neighbor matching was conducted using a caliper width of 0.1 and without replacement. Standardized mean differences (SMDs) less than 0.1 were considered indicative of adequate covariate balance.

Time-to-event outcomes were evaluated using Kaplan-Meier estimates and Cox proportional hazards regression models to calculate hazard ratios (HRs) with 95% CIs. Proportional hazards assumptions were tested using Schoenfeld residuals (eTable 4 in Supplement 1). The log-rank test was used to compare survival curves. Incidence rates per 1000 person-years were calculated by dividing the number of new events by the estimated total person-years at risk. The total person-years at risk were estimated by multiplying the total number of patients in each group by the mean follow-up time provided by the TriNetX platform for that group. All tests were 2-sided, with statistical significance set at *P* < .05. Analyses were conducted using the TriNetX platform and Stata, version 18 (StataCorp LLC).

To assess the robustness of our findings, we conducted multiple sensitivity analyses. First, we restricted the cohort to patients who initiated AOMs within 5 years from the date of the bariatric surgery to ensure temporal alignment. Second, a 6-month landmark analysis was performed to minimize the influence of early events unlikely to be associated with AOM exposure. Third, follow-up was extended to 3 years to evaluate potential delayed associations of bariatric surgery and AOMs with AUD. Fourth, we restricted the non-IBT group to phentermine users to reduce misclassification bias. Fifth, we limited the cohort to patients with 3 or more AOM prescriptions to reflect sustained treatment exposure.

## Results

### Baseline Characteristics

A total of 15 382 adult patients who received AOMs after bariatric surgery were included; 11 194 received IBT (mean [SD] age, 51.4 [11.6] years; 8855 women [79.1%] and 2339 men [20.9%]; 114 Asian [1.0%], 2394 Black or African American [21.4%], 1245 Hispanic or Latino [11.1%], 6895 White [61.6%], and 546 other race or ethnicity [4.8%]) and 4188 patients received non-IBT (mean [SD] age, 45.1 [11.0] years; 3587 women [86.6%] and 601 men [14.4%]; 20 Asian [0.4%], 990 Black or African American [23.6%], 621 Hispanic or Latino [14.8%], 2332 White [55.7%], and 225 other race or ethnicity [4.8%]). Individuals with preexisting AUD and those who received both IBT and non-IBT agents during follow-up were excluded (Table 1). A total of 4337 individuals who received IBT (38.7%) and 1682 individuals in the non-IBT group (40.2%) had a history of Roux-en-Y gastric bypass. At the index date of AOM initiation, 92.2% of the cohort (10 323 of 11 194) was within obesity class II or III. Semaglutide was the most frequent medication prescribed in the IBT group (9372 [83.7%]). In the non-IBT group, phentermine was the most frequent medication prescribed (3829 [91.4%]). The median time from bariatric surgery to AOM initiation was 3.7 years (IQR, 2.1-6.5 years) in the IBT group and 3.4 years (IQR, 1.9-6.2 years) in the non-IBT group. The median follow-up from the index date of AOM initiation to outcome was 2.0 years (IQR, 1.6-2.4 years) in both groups. Baseline patient characteristics are summarized in Table 1; additional details, including *P* values and density plots before and after matching, are provided in eTable 3 and eFigure 2 in Supplement 1.

**Table 1.** Baseline Characteristics of Patients Before and After PSM

Variable	Before PSM			After PSM		
	IBT (n = 11 194)	Non-IBT (n = 4188)	SMD	IBT (n = 3990)	Non-IBT (n = 3990)	SMD
Demographics						
Age at index, mean (SD), y	51.4 (11.6)	45.1 (11.0)	0.56	45.9 (11.0)	45.7 (10.8)	0.02
Sex, No. (%)						
Female	8855 (79.1)	3587 (86.6)	0.17	3387 (84.9)	3403 (85.3)	0.01
Male	2339 (20.9)	601 (14.4)	0.17	603 (15.1)	587 (14.7)	0.01
Race and ethnicity, No. (%)						
Asian	114 (1.0)	20 (0.4)	0.06	17 (0.4)	20 (0.5)	0.01
Black or African American	2394 (21.4)	990 (23.6)	0.05	940 (23.6)	937 (23.5)	<0.01
Hispanic or Latino	1245 (11.1)	621 (14.8)	0.11	567 (14.2)	545 (13.7)	0.02
White	6895 (61.6)	2332 (55.7)	0.12	2241 (56.2)	2265 (56.8)	0.01
Other^a^	546 (4.8)	225 (4.8)	0.04	225 (5.6)	223 (5.6)	0.02
Cardiometabolic factors						
Overweight and obesity, No. (%)	10 901 (97.4)	4001 (95.5)	0.10	3836 (96.1)	3821 (95.8)	0.02
BMI, mean (SD)	39.2 (7.9)	37 (7.2)	0.29	38.8 (7.7)	37.7 (7.2)	0.15
BMI ≥35, No. (%)	10 323 (92.2)	3796 (90.6)	0.07	3631 (91.0)	3631 (90.7)	0.01
Type 2 diabetes, No. (%)	6163 (55.1)	1091 (26.1)	0.62	1056 (26.5)	1088 (27.3)	0.02
HbA_1c_, mean (SD), %	6.2 (1.5)	5.5 (1.1)	0.53	5.6 (1.1)	5.5 (1.1)	0.09
HbA_1c_ ≥6.5%, No. (%)	4608 (41.2)	544 (13.0)	0.67	519 (13.0)	539 (13.5)	0.01
Hypertensive diseases, No. (%)	8177 (73.0)	2185 (52.2)	0.44	2173 (54.5)	2164 (54.2)	<0.01
Systolic blood pressure, mean (SD), mm Hg	127 (16.8)	124 (15.2)	0.21	125 (16.3)	124 (15.2)	0.06
Socioeconomic and psychosocial factors, No. (%)						
Problems related to education and literacy	25 (0.2)	10 (0.2)	<0.01	10 (0.3)	10 (0.3)	<0.01
Problems related to employment and unemployment	127 (1.1)	32 (0.8)	0.04	26 (0.7)	30 (0.8)	0.01
Problems related to housing and economic circumstances	174 (1.6)	31 (0.7)	0.08	35 (0.9)	31 (0.8)	0.01
Problems related to social environment	80 (0.7)	10 (0.2)	0.07	10 (0.3)	10 (0.3)	<0.01
Problems related to upbringing	86 (0.8)	28 (0.7)	0.01	25 (0.6)	27 (0.7)	<0.01
Other problems related to primary support group, including family circumstances	370 (3.3)	99 (2.4)	0.06	95 (2.4)	98 (2.5)	<0.01
Problems related to certain psychosocial circumstances	38 (0.3)	35 (0.8)	0.07	24 (0.6)	27 (0.7)	<0.01
Problems related to other psychosocial circumstances	237 (2.1)	70 (1.7)	0.03	68 (1.7)	67 (1.7)	<0.01
Psychiatric and substance abuse disorders, No. (%)						
Major depressive disorder	2132 (19.0)	637 (15.2)	0.10	639 (16.0)	618 (15.5)	0.01
Anxiety disorders	5847 (52.2)	1933 (46.2)	0.12	1938 (48.6)	1891 (47.4)	0.02
Bipolar disorder	603 (5.4)	166 (4.0)	0.07	178 (4.5)	163 (4.1)	0.02
Schizophrenia	64 (0.6)	12 (0.3)	0.04	16 (0.4)	12 (0.3)	0.02
Nicotine dependence	1437 (12.8)	503 (12.0)	0.03	473 (11.9)	472 (11.8)	<0.01
Cannabis-related disorders	182 (1.6)	64 (1.5)	<0.01	50 (1.3)	60 (1.5)	0.02
Cocaine-related disorders	60 (0.5)	14 (0.3)	0.03	15 (0.4)	14 (0.4)	<0.01
Stimulant-related disorders	53 (0.5)	12 (0.3)	0.03	12 (0.3)	12 (0.3)	<0.01
Sedative-, hypnotic-, or anxiolytic-related disorders	38 (0.3)	10 (0.2)	0.02	10 (0.3)	10 (0.3)	<0.01
Opioid-related disorders	295 (2.6)	65 (1.6)	0.08	63 (1.6)	65 (1.6)	<0.01
Off-label MAUD use, No. (%)						
Gabapentin	5795 (51.8)	1857 (44.3)	0.15	1797 (45.0)	1796 (45.0)	<0.01
Topiramate	3023 (27.0)	1126 (26.9)	<0.01	1120 (28.1)	1094 (27.4)	0.01
Baclofen	830 (7.4)	215 (5.1)	0.09	210 (5.3)	215 (5.4)	<0.01
Liver disease and laboratory test values						
Fibrosis and cirrhosis of liver, No. (%)	341 (3.0)	83 (2.0)	0.07	82 (2.1)	82 (2.1)	<0.01
Fatty (change of) liver, not elsewhere classified, No. (%)	2872 (25.7)	769 (18.4)	0.18	739 (18.5)	757 (19.0)	0.01
Chronic viral hepatitis, No. (%)	86 (0.8)	25 (0.6)	0.02	27 (0.7)	23 (0.6)	0.01
AST, mean (SD), U/L	23.0 (49.9)	21.7 (16.2)	0.03	21.8 (17.4)	21.8 (16.4)	<0.01
ALT, mean (SD), U/L	24.0 (38.6)	22.0 (20.3)	0.06	22.0 (21.8)	21.9 (19.8)	<0.01
ALP, mean (SD), U/L	84.7 (31.0)	81.6 (30.1)	0.10	80.8 (28.0)	81.6 (30.0)	0.03
Total bilirubin, mean (SD), mg/dL	0.5 (0.3)	0.5 (0.3)	0.02	0.5 (0.3)	0.5 (0.3)	0.01
INR, mean (SD)	1.1 (0.3)	1.1 (0.2)	0.10	1.1 (0.2)	1.1 (0.2)	<0.01
Platelets, mean (SD), ×10^9^/L	268.0 (75.4)	272.0 (69.1)	0.05	276.0 (73.1)	271.0 (69.4)	0.07
Albumin, mean (SD), g/dL	4.1 (0.4)	4.1 (0.4)	0.07	4.1 (0.4)	4.1 (0.4)	0.02

Abbreviations: ALP, alkaline phosphatase; ALT, alanine aminotransferase; AST, aspartate aminotransferase; BMI, body mass index (calculated as weight in kilograms divided by height in meters squared); HbA_1c_, hemoglobin A_1c_; IBT, incretin-based therapy; INR, international normalized ratio; MAUD, medication for alcohol use disorder; PSM, propensity score matching; SMD, standardized mean difference.

SI conversion factors: To convert albumin to grams per liter, multiply by 10.0; bilirubin to micromoles per liter, multiply by 17.104; AST, ALT, and ALP to microkatals per liter, multiply by 0.0167; and platelets to ×10^9^ per liter, multiply by 1.0.

^a^ Defined internally by TriNetX and includes American Indian or Alaska Native, Native Hawaiian or Other Pacific Islander, and all other nonspecified race or ethnic group categories.

Before PSM, patients in the IBT group were older, had a lower proportion of females, and exhibited higher rates of cardiometabolic risk factors (BMI ≥35, type 2 diabetes, and hypertension), psychiatric comorbidities (depression and anxiety), and liver disease (hepatic steatosis). After PSM, 3990 patients were included in each group. Residual imbalance was observed, with the IBT group having a higher mean (SD) BMI than the non-IBT group (38.8 [7.7] vs 37.7 [7.2]; SMD, 0.15) (Table 1).

### Outcomes

**Figure 1.**
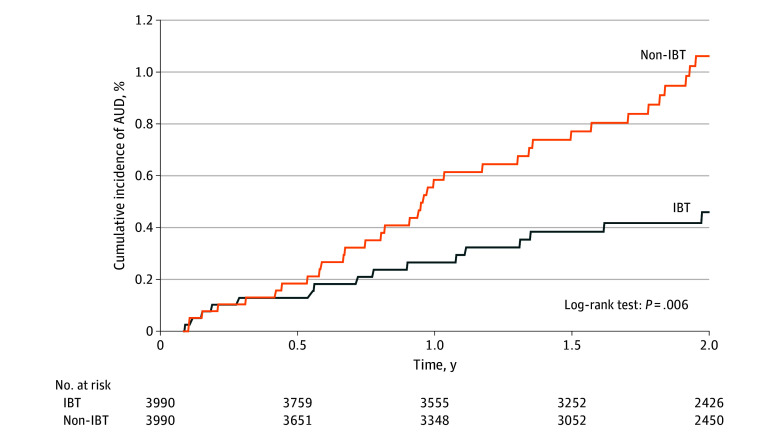
Cumulative Incidence of Alcohol Use Disorder (AUD) From Initiation of Antiobesity Medications After Bariatric Surgery IBT indicates incretin-based therapy.

A new diagnosis of AUD occurred for 16 patients in the IBT group and 35 patients in the non-IBT group, corresponding to incidence rates of 2.4 and 5.2 per 1000 person-years, respectively (Table 2). In time-to-event analyses, IBT was associated with a 55% lower hazard of developing AUD (HR, 0.45; 95% CI, 0.25-0.81; log-rank *P* = .006) (Table 2 and Figure 1).

**Table 2.** Events, Incidence Rate, and HR of developing AUD and Initiating MAUD at 2-Year Follow-Up Among Patients Undergoing Bariatric Surgery Prescribed IBT vs Non-IBT

Outcome	Events, No.		Incidence rate, per 1000 person-years		HR (95% CI)	*P* value
	IBT	Non-IBT	IBT	Non-IBT		
AUD	16	35	2.4	5.2	0.45 (0.25-0.81)	.006
MAUD	103	171	15.2	25.6	0.59 (0.46-0.75)	<.001

Abbreviations: AUD, alcohol use disorder; HR, hazard ratio, IBT, incretin-based therapy; MAUD, medication for alcohol use disorder.

**Figure 2.**
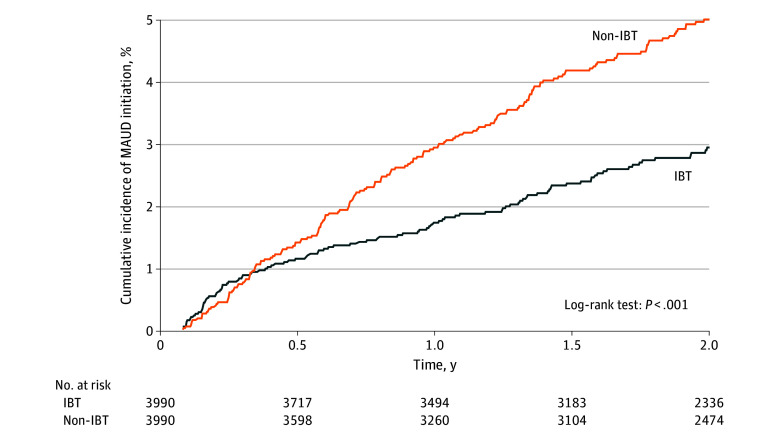
Cumulative Incidence of Medication for Alcohol Use Disorder (MAUD) Initiation From Initiation of Antiobesity Medications After Bariatric Surgery IBT indicates incretin-based therapy.

Initiation of MAUDs was observed for 103 patients in the IBT group and 171 patients in the non-IBT group, corresponding to incidence rates of 15.2 and 25.6 per 1000 person-years, respectively (Table 2). In time-to-event analyses, IBT was associated with a 41% lower hazard of initiating MAUDs (HR, 0.59; 95% CI, 0.46-0.75; log-rank *P* < .001) (Table 2 and Figure 2).

### Sensitivity Analysis

Findings remained consistent across multiple sensitivity analyses (Table 3). When the cohort was restricted to patients who initiated AOMs within 5 years of bariatric surgery, IBT was associated with a significantly lower risk of both AUD (HR, 0.43; 95% CI, 0.22-0.86; *P* = .01) and initiation of MAUDs (HR, 0.64; 95% CI, 0.49-0.83; *P* < .001). In a 6-month landmark analysis to mitigate the association of early events, IBT continued to be associated with a significantly lower risk of AUD (HR, 0.36; 95% CI, 0.18-0.72; *P* < .001) and initiation of MAUDs (HR, 0.63; 95% CI, 0.48-0.82; *P* < .001). With extended follow-up, the associations persisted; at 3 years, the risk of AUD was reduced (HR, 0.44; 95% CI, 0.25-0.74; *P* < .001), and the risk of initiating MAUDs remained significantly lower (HR, 0.71; 95% CI, 0.57-0.88; *P* = .002).

**Table 3.** Sensitivity Analyses Assessing the Events, Incidence Rate, and HR of Developing AUD and Initiating MAUD Among Patients Undergoing Bariatric Surgery Prescribed IBT vs Non-IBT

Sensitivity analysis outcome	Events, No.		Incidence rate, per 1000 person-years		HR (95% CI)	*P* value
	IBT	Non-IBT	IBT	Non-IBT		
≤5 y From bariatric surgery to AOMs						
AUD	12	27	2.5	5.6	0.43 (0.22-0.86)	.01
MAUD	90	138	18.5	28.7	0.64 (0.49-0.83)	<.001
6-mo Landmark						
AUD	11	30	1.6	4.5	0.36 (0.18-0.72)	<.001
MAUD	89	138	13.1	20.5	0.63 (0.48-0.82)	<.001
At 3 y						
AUD	19	45	2.3	5.1	0.44 (0.25-0.74)	<.001
MAUD	146	211	17.5	24.0	0.71 (0.57-0.88)	.002
Non-IBT (phentermine)						
AUD	15	32	2.3	5.0	0.46 (0.25-0.85)	.01
MAUD	105	156	16.2	24.5	0.66 (0.52-0.84)	<.001
≥3 AOM prescriptions						
AUD	≤10^a^	26	2.2	5.7	0.38 (0.18-0.79)	.007
MAUD	81	123	17.5	26.7	0.65 (0.49-0.85)	.002

Abbreviations: AOM, antiobesity medication; AUD, alcohol use disorder; HR, hazard ratio; IBT, incretin-based therapy; MAUD, medication for alcohol use disorder.

^a^ Values of 10 or less and corresponding incidence rates are suppressed for privacy.

When restricting the non-IBT group to phentermine users, IBT remained associated with a significantly lower risk of AUD (HR, 0.46; 95% CI, 0.25-0.85; *P* = .01) and initiation of MAUDs (HR, 0.66; 95% CI, 0.52-0.84; *P* < .001) (Table 3). Similarly, among patients who received at least 3 AOM prescriptions, IBT continued to show a protective association against AUD (HR, 0.38; 95% CI, 0.18-0.79; *P* = .007) and initiation of MAUDs (HR, 0.65; 95% CI, 0.49-0.85; *P* = .002).

## Discussion

In this large, multi-institutional cohort of patients who underwent bariatric surgery, the use of IBTs was associated with a 55% lower risk of new-onset AUD and a 41% lower risk of initiation of MAUDs compared with non-IBT AOMs. These associations were consistent across multiple sensitivity analyses, including a 6-month landmark analysis, restriction to patients initiating medications within 5 years of surgery, limiting analyses to sustained users, and restricting the comparator to phentermine.

Neurobiological and behavioral mechanisms may underlie these associations. In rodent models, GLP-1RAs are consistently associated with reduced alcohol intake and preference.^23,24^ Furthermore, repeated GLP-1RA administration was associated with reduced alcohol-associated reward learning and voluntary alcohol-seeking behavior, with effects most pronounced among animals consuming high amounts of alcohol.^25,26^ These effects are mediated through GLP-1 receptors located in key reward centers, such as the ventral tegmental area and nucleus accumbens, where GLP-1RAs attenuate dopaminergic signaling linked to alcohol reinforcement.^27,28,29^ Observational studies in humans also suggest protective associations between IBTs and alcohol-related-harm.^16,18,30^ In a randomized clinical trial, exenatide reduced heavy drinking days and total alcohol intake among participants with obesity and AUD.^31^ A secondary analysis of another trial found that dulaglutide was associated with a 29% reduction in alcohol consumption compared with placebo after 12 weeks.^15^ More recently, a phase 2 trial of semaglutide (0.5 mg weekly) demonstrated significant reductions in craving, alcohol consumption in a posttreatment laboratory self-administration task, drinks per drinking day, and heavy drinking days compared with placebo, although no significant change was observed in drinks per calendar day.^32^

The novelty of our study lies in evaluating IBTs within a population at heightened risk of hazardous alcohol consumption.^4,6,33,34,35^ Patients who have undergone bariatric surgery experience altered alcohol pharmacokinetics, with faster absorption and higher peak concentrations, amplifying reward signaling and increasing susceptibility to AUD.^36,37^ Incretin-based therapies may counteract these effects by blunting the reinforcing properties of alcohol and potentially diminishing the substitution of alcohol for food, a behavioral mechanism frequently reported after bariatric surgery.^38^ We specifically evaluated patients with inadequate weight loss after bariatric surgery, as demonstrated by the persistently elevated BMI at the index date of AOM initiation, with more than 90% of the cohort remaining within obesity class II or III. This subgroup of patients who have undergone bariatric surgery retains sustained cardiometabolic risk factors and has an even greater predisposition to liver disease when combined with the risk of increased alcohol consumption. In this population, IBTs may be associated with mitigated risk by serving as effective antiobesity therapy and by attenuation of alcohol craving. The median time from bariatric surgery to initiation of AOMs was 3.7 years in the IBT group and 3.4 years in the non-IBT group, coinciding with the period of greatest risk for new-onset AUD, as demonstrated by a systematic review and meta-analysis showing a significant and sustained increase in alcohol use beginning at 2 years postoperatively.^39^ Incorporating IBTs into post–bariatric surgery management could therefore represent a strategy to reduce long-term morbidity. Given the elevated AUD risk among patients who have undergone bariatric surgery, clinicians should consider prioritizing IBTs over non-IBT AOMs for patients with inadequate weight loss or AUD risk factors, complementing the American Society for Metabolic and Bariatric Surgery recommendations for AUD screening and counseling.^9^

### Strengths and Limitations

Our sensitivity analysis strengthens the validity of these results through multiple key findings. First, the protective association of IBT against both AUD and MAUD initiation was consistent across all analytic windows, from the 6-month landmark analysis through the 3-year follow-up. This persistent association over time suggests that IBT’s benefits could be maintained regardless of whether treatment is initiated early or later in the postoperative period, with a modestly stronger association observed closer to the time of bariatric surgery. Although this has not, to our knowledge, been directly studied after bariatric surgery, prior work has shown that the timing of IBT initiation does not change the outcome.^33^ Overall, the balance of evidence supports sustained IBT responsiveness of reward circuitry over time. Second, the sensitivity analysis showed that the protective association of IBT persisted when compared with phentermine users and patients receiving multiple AOMs. This interpretation is consistent with preclinical data showing that phentermine does not selectively reduce alcohol consumption.^40^

Several limitations have been encountered in this study. First, it is a retrospective observational analysis based on diagnostic and procedure billing codes, which may be subject to misclassification. To address this, we used both diagnostic codes for AUD, which are highly specific, and prescriptions for MAUDs, which are more sensitive, to identify the primary outcome. This approach explains the discrepancy in the number of events between the 2 outcomes. Second, AUD-specific psychotherapy was poorly captured, likely reflecting underuse of AUD-specific psychotherapy codes; this precluded its inclusion as a treatment outcome. Third, behavioral factors such as alcohol consumption patterns, binge drinking, and quantity of intake were not captured and may represent unmeasured confounding. We attempted to address this by matching on proxies for risk-taking behavior, including history of substance abuse and psychiatric comorbidities, as well as baseline liver enzyme levels and synthetic function markers. Fourth, the primary analysis was limited to a 2-year follow-up to ensure comparable median follow-up times between groups, given the more recent approval of IBTs as AOMs. Fifth, residual imbalance in BMI persisted after PSM, likely reflecting preferential prescribing of IBTs to patients with higher BMI who were considered candidates for more effective long-term AOM therapy. Nevertheless, BMI is unlikely to confound the association between bariatric surgery and AUD risk, as it is primarily related to metabolic rather than neurobehavioral outcomes. Furthermore, more than 90% of patients in both groups were classified as obesity class II or III, making this residual imbalance of marginal clinical significance. Sixth, generalizability may be limited, as the study population was restricted to individuals who required AOMs after bariatric surgery due to inadequate weight loss or weight regain.

## Conclusions

In this large, multi-institutional cohort of patients who underwent bariatric surgery, IBT was associated with a substantially lower risk of developing AUD and initiating MAUDs compared with non-IBT AOMs, with consistent findings across sensitivity analyses. These results suggest that, beyond weight loss efficacy, IBTs may be associated with neurobehavioral benefits that reduce alcohol-related harm. For patients who have undergone bariatric surgery, particularly those with inadequate weight loss or heightened AUD risk, clinicians should consider prioritizing IBTs over other AOMs. Prospective studies should confirm these findings, evaluate IBTs’ durability beyond 2 years, and examine their association with alcohol-related liver disease.

## Notes

Supplement 1. **eFigure 1.** Flow Diagram of Study Cohort Selection **eFigure 2.** Propensity Score Density for Bariatric Surgery Patients Who Received IBT vs Non-IBT Before and After PSM **eTable 1.** Diagnoses, Medications, and Procedural Codes Used in Constructing the Cohort and Defining the Outcomes **eTable 2.** Covariate Definitions and Corresponding Characteristic IDs (LOINC Codes, *ICD-10* Codes, and Other Standardized Identifiers) Used for Propensity Score Matching **eTable 3.** Baseline Characteristics Before and After PSM With *P* Values **eTable 4.** Schoenfeld Residual Test Results for Proportional Hazards Assumption Across Analyses

Supplement 2.
Data Sharing Statement
