# Supplementary material for: Incretin-Based Therapies and Post–Bariatric Surgery Alcohol Use Disorder
Source: JAMA Netw Open. 2025 Dec 22;8(12):e2549086. doi: 10.1001/jamanetworkopen.2025.49086 (PMC12723548; doi:10.1001/jamanetworkopen.2025.49086)
Supplement: Supplement 1. — eFigure 1. Flow Diagram of Study Cohort Selection eFigure 2. Propensity Score Density for Bariatric Surgery Patients Who Received IBT vs Non-IBT Before and After PSM eTable 1. Diagnoses, Medications, and Procedural Codes Used in Constructing the Cohort and Defining the Outcomes eTable 2. Covariate Definitions and Corresponding Characteristic IDs (LOINC Codes, ICD-10 Codes, and Other Standardized Identifiers) Used for Propensity Score Matching eTable 3. Baseline Characteristics Before and After PSM With P Values eTable 4. Schoenfeld Residual Test Results for Proportional Hazards Assumption Across Analyses [file jamanetwopen-e2549086-s001.pdf]

Supplementary Online Content

Fakhoury B, Sierra L, Rama K, Jahagirdar V, Díaz LA, Arab JP. Incretin-based therapies and post–bariatric surgery alcohol use disorder. *JAMA Netw Open.* 2025;8(12):e2549086. doi:10.1001/jamanetworkopen.2025.49086

eFigure 1. Flow Diagram of Study Cohort Selection

eFigure 2. Propensity Score Density for Bariatric Surgery Patients Who Received IBT vs Non-IBT Before and After PSM

eTable 1. Diagnoses, Medications, and Procedural Codes Used in Constructing the Cohort and Defining the Outcomes

eTable 2. Covariate Definitions and Corresponding Characteristic IDs (LOINC Codes, *ICD-10* Codes, and Other Standardized Identifiers) Used for Propensity Score Matching

eTable 3. Baseline Characteristics Before and After PSM With *P* Values

eTable 4. Schoenfeld Residual Test Results for Proportional Hazards Assumption Across Analyses

This supplementary material has been provided by the authors to give readers additional information about their work.

© 2025 Fakhoury B et al. *JAMA Network Open.*

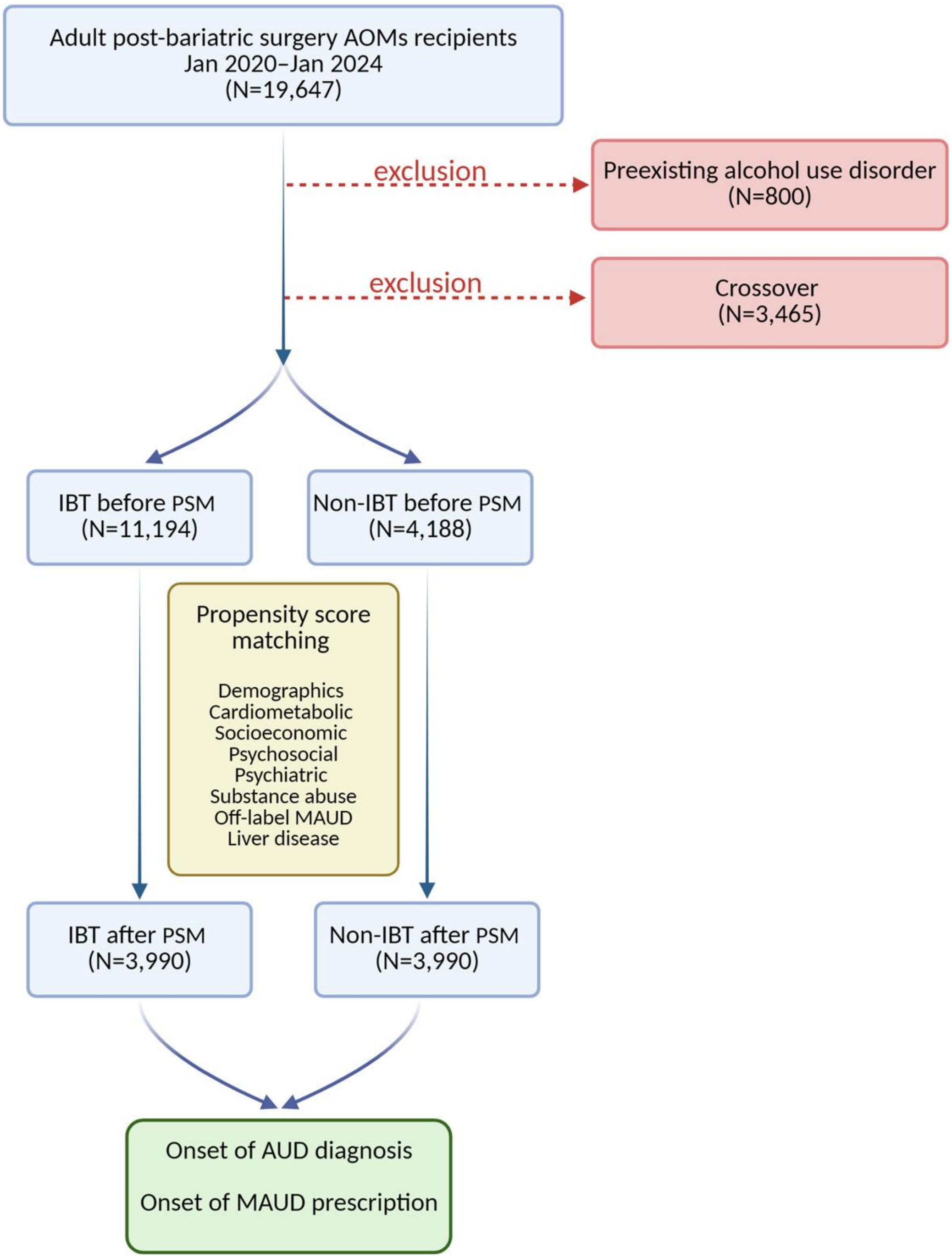

eFigure 1. Flow Diagram of Study Cohort Selection. Abbreviations: AUD, alcohol use disorder; IBT, incretin-based therapy; MAUD, medications for alcohol use disorder; PSM, propensity score matching.

© 2025 Fakhoury B et al. *JAMA Network Open.*

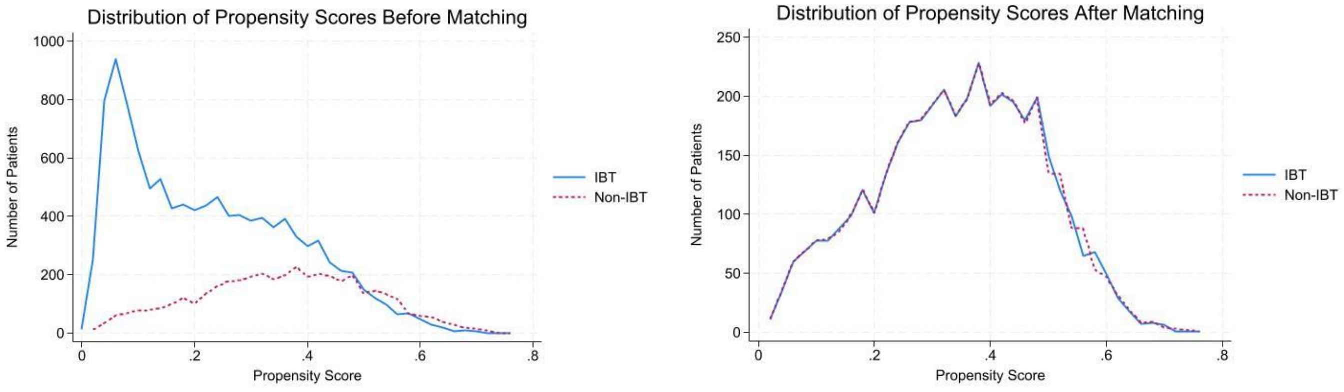

eFigure 2. Propensity Score Density for Bariatric Surgery Patients Who Received IBT vs Non-IBT Before and After PSM. Abbreviations: IBT, incretin-based therapy.

© 2025 Fakhoury B et al. *JAMA Network Open.*

eTable 1. Diagnoses, Medications, and Procedural Codes Used in Constructing the Cohort and Defining the Outcomes

Variable	Code	Definition
Bariatric surgery	UMLS:CPT:43775	Laparoscopy, surgical, gastric restrictive procedure; longitudinal gastrectomy (ie, sleeve gastrectomy)
	UMLS:CPT:43644	Laparoscopy, surgical, gastric restrictive procedure; with gastric bypass and Roux-en-Y gastroenterostomy (roux limb 150 cm or less)
	UMLS:CPT:43843	Gastric restrictive procedure, without gastric bypass, for morbid obesity; other than vertical-banded gastroplasty
	UMLS:CPT:43846	Gastric restrictive procedure, with gastric bypass for morbid obesity; with short limb (150 cm or less) Roux-en-Y gastroenterostomy
	UMLS:CPT:43847	Gastric restrictive procedure, with gastric bypass for morbid obesity; with small intestine reconstruction to limit absorption
	UMLS:CPT:43842	Gastric restrictive procedure, without gastric bypass, for morbid obesity; vertical-banded gastroplasty
	UMLS:CPT:43659	Unlisted laparoscopy procedure, stomach
	UMLS:CPT:43645	Laparoscopy, surgical, gastric restrictive procedure; with gastric bypass and small intestine reconstruction to limit absorption
	UMLS:ICD10PCS:0D190Z9	Bypass Duodenum to Duodenum, Open Approach
	UMLS:ICD10PCS:0DB60ZZ	Excision of Stomach, Open Approach
	UMLS:ICD10PCS:0D16479	Bypass Stomach to Duodenum with Autologous Tissue Substitute, Percutaneous Endoscopic Approach
	UMLS:ICD10PCS:0D1647A	Bypass Stomach to Jejunum with Autologous Tissue Substitute, Percutaneous Endoscopic Approach
	UMLS:ICD10PCS:0D164J9	Bypass Stomach to Duodenum with Synthetic Substitute, Percutaneous Endoscopic Approach
	UMLS:ICD10PCS:0D164JA	Bypass Stomach to Jejunum with Synthetic Substitute, Percutaneous Endoscopic Approach

© 2025 Fakhoury B et al. *JAMA Network Open.*

	UMLS:ICD10PCS:0D164K9	Bypass Stomach to Duodenum with Nonautologous Tissue Substitute, Percutaneous Endoscopic Approach
	UMLS:ICD10PCS:0D164KA	Bypass Stomach to Jejunum with Nonautologous Tissue Substitute, Percutaneous Endoscopic Approach
	UMLS:ICD10PCS:0DB64ZZ	Excision of Stomach, Percutaneous Endoscopic Approach
	UMLS:ICD10PCS:0DB80ZZ	Excision of Small Intestine, Open Approach
	UMLS:ICD10PCS:0DB64Z3	Excision of Stomach, Percutaneous Endoscopic Approach, Vertical
	UMLS:ICD10PCS:0DB63ZZ	Excision of Stomach, Percutaneous Approach
	UMLS:ICD10PCS:0DB67ZZ	Excision of Stomach, Via Natural or Artificial Opening
	UMLS:ICD10PCS:0D160ZA	Bypass Stomach to Jejunum, Open Approach
	UMLS:ICD10PCS:0D1607A	Bypass Stomach to Jejunum with Autologous Tissue Substitute, Open Approach
	UMLS:ICD10PCS:0D160JA	Bypass Stomach to Jejunum with Synthetic Substitute, Open Approach
	UMLS:ICD10PCS:0D160KA	Bypass Stomach to Jejunum with Nonautologous Tissue Substitute, Open Approach
	UMLS:ICD10PCS:0D168ZA	Bypass Stomach to Jejunum, Via Natural or Artificial Opening Endoscopic
	UMLS:ICD10PCS:0D168JA	Bypass Stomach to Jejunum with Synthetic Substitute, Via Natural or Artificial Opening Endoscopic
	UMLS:ICD10PCS:0D1687A	Bypass Stomach to Jejunum with Autologous Tissue Substitute, Via Natural or Artificial Opening Endoscopic
	UMLS:ICD10PCS:0D168KA	Bypass Stomach to Jejunum with Nonautologous Tissue Substitute, Via Natural or Artificial Opening Endoscopic
	UMLS:ICD10PCS:0D164ZA	Bypass Stomach to Jejunum, Percutaneous Endoscopic Approach

© 2025 Fakhoury B et al. *JAMA Network Open.*

	UMLS:ICD10PCS:0D164Z9	Bypass Stomach to Duodenum, Percutaneous Endoscopic Approach
	UMLS:ICD10PCS:0D160Z9	Bypass Stomach to Duodenum, Open Approach
	UMLS:ICD10PCS:0D168J9	Bypass Stomach to Duodenum with Synthetic Substitute, Via Natural or Artificial Opening Endoscopic
	UMLS:ICD10PCS:0D16079	Bypass Stomach to Duodenum with Autologous Tissue Substitute, Open Approach
	UMLS:ICD10PCS:0D160J9	Bypass Stomach to Duodenum with Synthetic Substitute, Open Approach
	UMLS:ICD10PCS:0D168Z9	Bypass Stomach to Duodenum, Via Natural or Artificial Opening Endoscopic
	UMLS:ICD10PCS:0D160K9	Bypass Stomach to Duodenum with Nonautologous Tissue Substitute, Open Approach
	UMLS:ICD10PCS:0D16879	Bypass Stomach to Duodenum with Autologous Tissue Substitute, Via Natural or Artificial Opening Endoscopic
	UMLS:ICD10PCS:0D168K9	Bypass Stomach to Duodenum with Nonautologous Tissue Substitute, Via Natural or Artificial Opening Endoscopic
History of Bariatric Surgery	UMLS:ICD10PCS:Z98.84	Bariatric surgery status
IBT	NLM:RXNORM:2601723	Tirzepatide
	NLM:RXNORM:1991302	Semaglutide
	NLM:RXNORM:475968	Liraglutide
Non-IBT		
	NLM:RXNORM:37925	Orlistat
	NLM:RXNORM:8152	Phentermine
	NLM:RXNORM:7243	Naltrexone (≤8mg)
	NLM:RXNORM:1422	Benzphetamine
	NLM:RXNORM:33272	Phendimetrazine
	NLM:RXNORM:3389	Diethylpropion
AUD	UMLS:ICD10PCS:F10.1	Alcohol abuse
	UMLS:ICD10PCS:F10.2	Alcohol dependence
MAUD	NLM:RXNORM:7243	Naltrexone (≥50mg)

© 2025 Fakhoury B et al. *JAMA Network Open.*

	NLM:RXNORM:82819	Acamprosate
	NLM:RXNORM:3554	Disulfiram

Abbreviations: AUD: Alcohol Use Disorder; IBT: Incretin-Based Therapy; MAUD: Medications for Alcohol Use Disorder.

© 2025 Fakhoury B et al. *JAMA Network Open.*

eTable 2. Covariate Definitions and Corresponding Characteristic IDs (LOINC Codes, *ICD-10* Codes, and Other Standardized Identifiers) Used for Propensity Score Matching

Covariate	Code
Age at Index	Variable:AI
White	UMLS:2106-3
Female	UMLS:F
Hispanic or Latino	UMLS:2135-2
Black or African American	UMLS:2054-5
Other Race	UMLS:2131-1
Asian	UMLS:2028-9
Nicotine dependence	ICD10CM:F17
Type 2 diabetes mellitus	ICD10CM:E11
Hypertensive diseases	ICD10CM:I10–I1A
Overweight and obesity	ICD10CM:E66
Cannabis-related disorders	ICD10CM:F12
Opioid-related disorders	ICD10CM:F11
Other stimulant-related disorders	ICD10CM:F15
Other anxiety disorders	ICD10CM:F41
Bipolar disorder	ICD10CM:F31
Schizophrenia	ICD10CM:F20
Chronic viral hepatitis	ICD10CM:B18
Major depressive disorder, recurrent	ICD10CM:F33
Cocaine-related disorders	ICD10CM:F14
Fatty liver, not elsewhere classified	ICD10CM:K76.0
Fibrosis and cirrhosis of liver	ICD10CM:K74
Sedative, hypnotic, or anxiolytic-related disorders	ICD10CM:F13
Problems related to education and literacy	ICD10CM:Z55
Problems related to employment/unemployment	ICD10CM:Z56

© 2025 Fakhoury B et al. *JAMA Network Open.*

Problems related to housing/economic circumstances	ICD10CM:Z59
Problems related to social environment	ICD10CM:Z60
Problems related to upbringing	ICD10CM:Z62
Problems related to primary support group	ICD10CM:Z63
Problems related to psychosocial circumstances	ICD10CM:Z64
Other psychosocial circumstances	ICD10CM:Z65
Baclofen	RXNORM:1292
Topiramate	RXNORM:38404
Gabapentin	RXNORM:25480
BMI	LOINC:9083
Blood Pressure, Systolic	LOINC:9085
Hemoglobin A1c	LOINC:9037
Alanine aminotransferase (ALT)	LOINC:9044
Aspartate aminotransferase (AST)	LOINC:9047
Alkaline phosphatase	LOINC:9046
Bilirubin, total	LOINC:9050
Albumin	LOINC:9045
Platelets	LOINC:9020
INR	LOINC:9032

Abbreviations: AI, age at index; ALP, alkaline phosphatase; ALT, alanine aminotransferase; AST, aspartate aminotransferase; BMI, body mass index; F, female; HbA1c, hemoglobin A1c; ICD-10-CM, International Classification of Diseases 10th Revision Clinical Modification; INR, international normalized ratio; LOINC, Logical Observation Identifiers Names and Codes; RxNorm, standardized drug vocabulary; UMLS, Unified Medical Language System.

© 2025 Fakhoury B et al. *JAMA Network Open.*

eTable 3. Baseline Characteristics Before and After PSM With *P* Values

Variable	Before PSM			After PSM		
	IBT (n=11,194)	Non-IBT (n=4,188)	p-value	IBT (n=3,990)	Non-IBT (n=3,990)	p-value
Demographics						
Age at Index, mean (SD), y	51.4 (11.6)	45.1 (11.0)	<0.001	45.9 (11.0)	45.7 (10.8)	0.37
Sex, Female, No. (%)	8,855 (79.1)	3,587 (86.6)	<0.001	3,387 (84.9)	3,403 (85.3)	0.61
Race and ethnicity, No. (%)						
Asian	114 (1.0)	20 (0.4)	<0.001	17 (0.4)	20 (0.5)	0.58
Black or African American	2,394 (21.4)	990 (23.6)	0.003	940 (23.6)	937 (23.5)	0.94
Hispanic or Latino	1,245 (11.1)	621 (14.8)	<0.001	567 (14.2)	545 (13.7)	0.48
White	6,895 (61.6)	2,332 (55.7)	0.001	2,241 (56.2)	2,265 (56.8)	0.45
Other[a]	546 (4.8)	225 (4.8)	0.04	17 (0.426%)	20 (0.501%)	0.62
Cardiometabolic Factors						
Overweight and obesity, No. (%)	10,901 (97.4)	4,001 (95.5)	<0.001	3,836 (96.1)	3,821 (95.8)	0.39
BMI, mean (SD)	39.2 (7.86)	37 (7.17)	<0.001	38.8 (7.72)	37.7 (7.19)	<0.001
BMI ≥, No. (%)	10,323 (92.2)	3,796 (90.6)	0.001	3,631 (91.0)	3,631 (90.7)	0.87
Type 2 diabetes mellitus, n (%)	6,163 (55.1)	1,091 (26.1)	<0.001	1,056 (26.5)	1,088 (27.3)	0.42
HbA1c, mean (SD), %	6.2 (1.5)	5.5 (1.1)	<0.001	5.6 (1.1)	5.5 (1.1)	<0.001
HbA1c≥6.5%, No. (%)	4,608 (41.2)	544 (13.0)	<0.001	519 (13.0)	539 (13.5)	0.41
Hypertensive diseases, No. (%)	8,177 (73.0)	2,185 (52.2)	<0.001	2,173 (54.5)	2,164 (54.2)	0.84
Blood Pressure, Systolic, mean (SD), mmHg	127 (16.8)	124 (15.2)	<0.001	125 (16.3)	124 (15.2)	0.02
Socioeconomic and Psychosocial Factors						
Problems related to education and literacy, No. (%)	25 (0.2)	10 (0.2)	0.85	10 (0.3)	10 (0.3)	0.99
Problems related to employment and unemployment, No. (%)	127 (1.1)	32 (0.8)	0.04	26 (0.7)	30 (0.8)	0.58
Problems related to housing and economic circumstances, No. (%)	174 (1.6)	31 (0.7)	<0.001	35 (0.9)	31 (0.8)	0.62
Problems related to social environment, No. (%)	80 (0.7)	10 (0.2)	<0.001	10 (0.3)	10 (0.3)	0.99
Problems related to upbringing, No. (%)	86 (0.8)	28 (0.7)	0.52	25 (0.6)	27 (0.7)	0.78
Other problems related to primary support group, including family circumstances, No. (%)	370 (3.3)	99 (2.4)	0.003	95 (2.4)	98 (2.5)	0.82
Problems related to certain psychosocial circumstances, No. (%)	38 (0.3)	35 (0.8)	<0.001	24 (0.6)	27 (0.7)	0.67
Problems related to other psychosocial circumstances, No. (%)	237 (2.1)	70 (1.7)	0.08	68 (1.7)	67 (1.7)	0.93

© 2025 Fakhoury B et al. *JAMA Network Open.*

Psychiatric and Substance Abuse Disorders						
Major depressive disorder, No. (%)	2,132 (19.0)	637 (15.2)	<0.001	639 (16.0)	618 (15.5)	0.52
Anxiety disorders, No. (%)	5,847 (52.2)	1,933 (46.2)	<0.001	1,938 (48.6)	1,891 (47.4)	0.29
Bipolar disorder, No. (%)	603 (5.4)	166 (4.0)	<0.001	178 (4.5)	163 (4.1)	0.41
Schizophrenia, No. (%)	64 (0.6)	12 (0.3)	0.02	16 (0.4)	12 (0.3)	0.45
Nicotine dependence, No. (%)	1,437 (12.8)	503 (12.0)	0.17	473 (11.9)	472 (11.8)	0.97
Cannabis related disorders, No. (%)	182 (1.6)	64 (1.5)	0.67	50 (1.3)	60 (1.5)	0.34
Cocaine related disorders, No. (%)	60 (0.5)	14 (0.3)	0.11	15 (0.4)	14 (0.4)	0.85
Stimulant related disorders, No. (%)	53 (0.5)	12 (0.3)	0.11	12 (0.3)	12 (0.3)	0.99
Sedative, hypnotic, or anxiolytic related disorders, No. (%)	38 (0.3)	10 (0.2)	0.31	10 (0.3)	10 (0.3)	0.99
Opioid related disorders, No. (%)	295 (2.6%)	65 (1.6%)	<0.001	63 (1.6)	65 (1.6)	0.86
Off-label MAUD						
Gabapentin, No. (%)	5,795 (51.8)	1,857 (44.3)	<0.001	1,797 (45.0)	1,796 (45.0)	0.98
Topiramate, No. (%)	3,023 (27.0)	1,126 (26.9)	0.88	1,120 (28.1)	1,094 (27.4)	0.52
Baclofen, No. (%)	830 (7.4)	215 (5.1)	<0.001	210 (5.3)	215 (5.4)	0.80
Liver disease and Laboratory test values						
Fibrosis and cirrhosis of liver, No. (%)	341 (3.0)	83 (2.0)	<0.001	82 (2.1)	82 (2.1)	0.41
Fatty (change of) liver, not elsewhere classified, No. (%)	2,872 (25.7)	769 (18.4)	<0.001	739 (18.5)	757 (19.0)	0.61
Chronic viral hepatitis, No. (%)	86 (0.8)	25 (0.6)	0.27	27 (0.7)	23 (0.6)	0.57
AST, mean (SD), U/L	23.0 (49.9)	21.7 (16.2)	0.12	21.8 (17.4)	21.8 (16.4)	0.94
ALT, mean (SD), U/L	24.0 (38.6)	22.0 (20.3)	0.002	22.0 (21.8)	21.9 (19.8)	0.90
ALP, mean (SD), U/L	84.7 (31.0)	81.6 (30.1)	<0.001	80.8 (28.0)	81.6 (30.0)	0.18
Total bilirubin, mean (SD), mg/dL	0.5 (0.3)	0.5 (0.3)	0.37	0.5 (0.3)	0.5 (0.3)	0.59
INR, mean (SD)	1.1 (0.3)	1.1 (0.2)	<0.001	1.1 (0.2)	1.1 (0.2)	0.87
Platelets, mean (SD), $\times 10^9$/L	268.0 (75.4)	272.0 (69.1)	0.01	276.0 (73.1)	271.0 (69.4)	0.003
Albumin, mean (SD), g/dL	4.1 (0.4)	4.1 (0.4)	<0.001	4.1 (0.4)	4.1 (0.4)	0.29

Abbreviations: ALP, alkaline phosphatase; ALT, alanine aminotransferase; AST, aspartate 1 aminotransferase; BMI, body mass index (calculated as weight in kilograms divided by height 2 in meters squared); HbA1c, hemoglobin A1c; IBT, incretin-based therapy; INR, international 3 normalized ratio; MAUD, medication for alcohol use disorder; PSM, propensity score 4 matching; SMD, standardized mean difference.

SI conversion factors: To convert albumin to grams per liter, multiply by 10.0; bilirubin to 6 micromoles per liter, multiply by 17.104; AST, ALT, and ALP to microkatals per liter, multiply 7 by 0.0167; and platelets to ×109 per liter, multiply by 1.0. 8

[a] Defined internally by TriNetX

© 2025 Fakhoury B et al. *JAMA Network Open.*

eTable 4. Schoenfeld Residual Test Results for Proportional Hazards Assumption Across Analyses

Analysis	Outcome	p-value
Primary Analysis	AUD	0.52
	MAUD	0.46
<5 years from BS to AOMs	AUD	0.42
	MAUD	0.77
6 months Landmark	AUD	0.89
	MAUD	0.40
At 3 years	AUD	0.37
	MAUD	0.25
Non-IBT (Phentermine)	AUD	0.48
	MAUD	0.54
3 Instances of AOMs	AUD	0.62
	MAUD	0.17

Abbreviations: AUD, alcohol use disorder; IBT, incretin-based therapy; MAUD, medications for alcohol use disorder.

© 2025 Fakhoury B et al. *JAMA Network Open.*
