# Supplementary material for: Incretin-Based Therapies and Post–Bariatric Surgery Alcohol Use Disorder
Source: JAMA Netw Open. 2025 Dec 22;8(12):e2549086. doi: 10.1001/jamanetworkopen.2025.49086 (PMC12723548; doi:10.1001/jamanetworkopen.2025.49086)
Supplement: Supplement 2. — Data Sharing Statement [file jamanetwopen-e2549086-s002.pdf]

Data Sharing Statement

Fakhoury. Incretin-Based Therapies and Post–Bariatric Surgery Alcohol Use Disorder. *JAMA Netw Open*. Published December 22, 2025. doi:10.1001/jamanetworkopen.2025.49086

Data
Data available: No

Additional Information
Explanation for why data not available: The data underlying this article are available through the TriNetX Research Network. Due to licensing restrictions, the authors are not permitted to share the data directly.
